# Pregnancy in women with spinal muscular atrophy (SMA): maternal and neonatal outcomes with multi-speciality management

**DOI:** 10.1007/s00415-026-13629-z

**Published:** 2026-01-20

**Authors:** David Cohen, Melanie Nana, Nicholas Hart, Lindsay Arrandale, Con Kelleher, Catherine Nelson-Piercy

**Affiliations:** 1 King’s College, London, UK; 2 Guy’s and St Thomas’s Hospital NHS Foundation Trust, London, UK; 3 Lane Fox Clinical Respiratory Physiology Research Centre, Guy’s and St Thomas’ NHS Foundation Trust, London, UK; 4 Centre for Human and Applied Physiological Sciences, King’s College London, London, UK

**Keywords:** Spinal muscular atrophy, Pregnancy, Pre-pregnancy counselling, SMA pregnancy

## Abstract

Spinal muscular atrophy (SMA) is a rare inherited disorder that results in skeletal muscle wasting and weakness with a varying degree of severity. Pregnancy is associated with several changes in respiratory muscle function and respiratory physiology, which can compromise breathing leading to complications during pregnancy and delivery. Pregnant women with SMA are therefore considered to be a high-risk obstetric group. Due to the rare nature of the condition, it is infrequently encountered in pregnancy and this highlights the clinical importance of reporting this current case series. We report, in this retrospective case series, the outcomes of eight pregnancies in six women living with SMA, including, to our knowledge, the first reported successful pregnancy in a woman with SMA type-1, managed in our tertiary multi-professional and multi-speciality centre. All pregnancies, over an 18-year period, resulted in healthy live births between 30 and 39 weeks of gestation, six were pre-term (before 37 weeks gestation) and two were term. Although there were no maternal deaths, four women had a deterioration in respiratory function during the second trimester. All, but one returned to their pre-pregnancy state by three months postpartum. One had an obstetric-related post-partum complication and returned to pre-pregnancy baseline by the first year postpartum. Our case series, of a rare neuromuscular condition in pregnancy, strongly supports that appropriate multi-professional and multi-speciality care for pregnant women living with SMA enhances the outcome for both mother and baby. Indeed, two of our women had the confidence to proceed with a second pregnancy, both of which concluded in good outcomes.

## Introduction

Spinal muscular atrophy (SMA) is a rare genetic disorder with an incidence of 1 in 10,000 live births [1]. The condition leads to progressive loss of motor neurons in the ventral horns of the spinal cord, resulting in skeletal muscle wasting and weakness, particularly impacting locomotor and respiratory muscles. Although there are four types, type 1 SMA rarely progresses beyond childhood, whereas types 2, 3 and 4 are less severe and more slowly progressive [2]. Furthermore, with targeted nutritional support and respiratory support, for example non-invasive ventilation (NIV) to manage chronic respiratory failure, combined with advances in gene therapy and novel drugs, such as antisense oligonucleotide inhibitors, the life expectancy of individuals with SMA is increasing, with women reaching childbearing age [3, 4]. We report the management, maternal and neonatal outcomes of six women across eight pregnancies from our multi-professional, multi-specialty maternity unit.

## Methods

A retrospective review of maternal and neonatal outcomes for all women living with SMA who received obstetric care at St Thomas’ Hospital in London between 2007–2025 was performed. Patients were identified from an anonymised database of women who received care under the obstetric medicine team. The multi-speciality team consists of an experienced obstetrician, obstetric medicine physician, obstetric anaesthetist and respiratory and critical care physician. All women identified were contacted and written informed consent was obtained for inclusion in this case series.

Maternal demographics, SMA type, functional status, maternal and neonatal outcomes, mode of delivery, gestation at delivery, anaesthetic details and neonatal outcomes were collected from hospital records. Where outcomes were not reported, patients were contacted via telephone. Primary outcomes of interest were maternal and neonatal morbidity and mortality as well as maternal respiratory deterioration. Secondary outcomes included pregnancy complications, mode of delivery, anaesthesia-related issues and neonatal health metrics.

Continuous variables were expressed as a mean ± standard deviation (SD) or median [interquartile range (IQR)], depending on normality of distribution of the data.

## Results

### Demographics and disease characteristics

Eight pregnancies in six women living with SMA were identified. Median maternal age at delivery was 29 ± 7 years. Six pregnancies were spontaneous, two were achieved with in vitro fertilisation (IVF)—both in the same woman, due to unexplained infertility. Demographics, including SMA type, are summarised in Table 1. Four out of the six women in our study have SMA type-2, one has SMA type-3, and one has SMA type-1. All women were wheelchair dependent prior to conception. Three women used home NIV pre-pregnancy. The woman with SMA type-1 who had not had a spinal fusion but had severe kyphoscoliosis and several fixed flexion deformities required modifications to her wheelchair to allow for growth of her pregnant abdomen.

**Table 1:** Patient demographics

Patient ID	Pregnancy	Age at delivery (years)	SMA type	Conception	PPC
1	1	27	2	Spontaneous	No
2	2	23	2	Spontaneous	Yes
3	3	36	3	Spontaneous	Yes
4	4	30	2	Spontaneous	Yes
3	5	33	3	Spontaneous	Yes
5	6	27	2	IVF	Yes
6	7	24	1	Spontaneous	No
5	8	34	2	IVF	Yes

*SMA* spinal muscular atrophy, *PPC* pre-pregnancy counselling (Pregnancies listed in chronological order)

### Maternal and pregnancy outcomes

Four of the women received pre-pregnancy counselling (PPC) involving discussion around the impact of SMA on maternal health during pregnancy. Of the two who did not, one had an unplanned pregnancy, the second only had genetic counselling regarding the risk of any offspring inheriting SMA. The three women who used home NIV pre-pregnancy experienced a decline in their respiratory status as pregnancy progressed, as expected, requiring an increase in duration of NIV and/or change in NIV settings (inspiratory positive airways pressure and back up rate up-titrated). All were weaned back to pre-pregnancy settings of NIV within one month of delivery. Median gestational age at birth was 35 weeks and five days [IQR 6-7 days]. All were delivered by elective caesarean section under general anaesthetic after assessment by the multi-speciality team. Three women required awake fibreoptic intubation. Tables 2 and 3 summarise the maternal outcomes.

**Table 2:** Maternal outcomes (Antenatal and Delivery)

Pregnancy	Respiratory support pre-pregnancy	Antenatal complications/admissions	Hours of NIV support in antenatal period	Mode of Delivery	Anaesthesia at delivery	Preterm labour/obstetric complication (PET, GDM, PPH)	Delivery complications
1	None	Pre-existing hypothyroidism	None	ELCS	GA	GDM—diet controlled	No—received prophylactic antibiotics to prevent chest infection
2	Night NIV 10–12 h per night, cough assist as required	postpartum LRTI	Night NIV 10-12h per night, 3–4 h in the afternoon from 19 weeks	ELCS	Awake fibreoptic intubation GA	No	No
3	None	influenza	overnight NIV from 33 weeks until delivery	ELCS	GA	No	Bowel injury leading to 4 week ITU admission, developed respiratory distress. Poor post-op surgical wound healing—VAC dressing for 4 months
4	None	LRTI at 35 weeks—admitted for 1 night—no NIV required	None	ELCS	Awake fibreoptic intubation GA	No	No
5	None	H1N1 infections × 2, ITU admission for pneumonia	NIV while inpatient with respiratory infection	ELCS	GA	No	No
6	Night NIV + cough assist—low adherence	1 st trimester chest infection—no admission	From 19 weeks NIV at night and as required during the day. Weekly cough-assist use in last trimester of pregnancy	ELCS	Awake fibreoptic intubation GA	No	No
7	NIV 17 h per day		Increase of NIV use to 24 h per day at 18 weeks	ELCS	GA	No	No
8	Night NIV + cough assist—low adherence	2nd trimester—gastroenteritis complicated by catheter-associated UTI	Used once at 23 weeks used overnight for two weeks	ELCS	Awake fibreoptic intubation GA	No	No

*NIV* non-invasive ventilation, *PRN* as required, *GA* general anaesthetic, *LRTI* lower respiratory tract infection, *UTI* urinary tract infection, *C/S* caesarean section, *ELCS* elective lower segment caesarean section, *PET* preeclampsia toxaemia, *GDM* gestational diabetes mellitus, *PPH* postpartum haemorrhage

**Table 3:** Maternal outcomes (Postnatal and Mortality)

Pregnancy	Respiratory support post-pregnancy	Progress since delivery	Long-term follow-up	Mortality
1	None	Stable—working full-time	Stable. Commenced Risdiplam 14 years post partum (for last 4 years)	No
2	Night NIV 10–11 h, cough assist PRN	Now has SPC due to urinary retention secondary to Risdiplam	Commenced Risdiplam 5 years post-delivery due to increased weakness	No
3	Extubated to NIV for 48 h, cough-assist device—CPAP trial, but did not use postnatally	C/S wound infection, sepsis with postnatal collection, laparotomy and bowel injury, 2 postnatal chest infections, perforated appendix 4 months postnatal	Returned to pre-pregnancy state	No
4	NIV immediately after C/S but stopped 4 h post-op. cough assist device daily for 2 weeks postnatal	Gradual postnatal increase in weakness. 1 year PN—returned to work, but was struggling with fatigue. Commenced NIV 7–8 h at night for 6 months. Next 6 months only when ill. Sleep study improved at 2-year postnatal review, stopped regular NIV	Stable	No
5	None	Decided for second pregnancy 3 years later	Risdiplam for last 2 years,	No
6	Continuous NIV for first 48 h postpartum, then back to intermittent use as required	Decided for second pregnancy 7 years later	Returned to pre-pregnancy state	No
7	Decrease of NIV use to 20–21 h per day. Weaned back to pre-pregnancy level 1 month postnatal	No changes in NIV use postpartum. NIV setting increased but usage time stayed the same	Stable	No
8	Continuous NIV for 6 h	UTI requiring admission 2 months postnatally	Returned to pre-pregnancy state	No

*NIV* non-invasive ventilation, *CPAP* continuous positive airway pressure, *C/S* caesarean section, *UTI* urinary tract infection, *SPC* suprapubic catheter

### Neonatal outcomes

Six of the eight babies were born pre-term. Mean birth weight was 2540 g ± 658 g. Four required admission to the neonatal intensive care unit (NICU), three with respiratory distress, and one was admitted with necrotising enterocolitis. All NICU admissions were of babies born pre-term. There were two neonatal readmissions. No babies died and we have confirmed all are still alive and thriving at the time of writing. There were no reported congenital abnormalities. Table 4 summarises the neonatal outcomes.

**Table 4:** Neonatal outcomes

Pregnancy	Antenatal steroids	Birth weight (grams)	Gestational age (weeks + days)	Apgar score (1/5/10 min)	Congenital anomalies	Respiratory distress syndrome	Neonatal intensive care unit (NICU) admission	Neonatal readmission?	Neonatal mortality
1	No	3401*	38 + 4	Not available	None	Required inflation breaths immediately after birth—recovered spontaneously	No	No	No
2	Yes	2940	36 + 4	4,6,10	None	no	Yes—2 days	No	No
3	Yes	3232	37 + 1	2, 4	None	no	No	No	
4	Yes	2580	36 + 2	Not available	None	None	NO	Apnoeas day 10 secondary to reflux—resolved with antacid	No
5	Yes	2580	35 + 0	2, 4	None	Yes	Three week admission—with RDS	No	No
6		2190	35 + 0	6, 6	None	No	no	No	No
7	No	1474	30 + 0	4,5	None	Yes	Admitted for three months with NEC	1 week post discharge developed bronchiolitis	No
8	Yes	1920	34 + 4	4,5	None	Yes (TTN) 2 × sets of inflation breaths, 1 × ventilation breaths given	Yes—5 days	No	No

*NICU* neonatal intensive care unit, *NEC* necrotising enterocolitis, *BW* birth weight, *RDS* respiratory distress syndrome, *TTN* transient tachypnoea of the neonate

## Discussion

For women living with SMA, embarking on a pregnancy may expose them to a significant risk of deterioration in their health [5], and presents unique challenges to medical teams managing them. Caring for these women is complex, and should be led by a highly skilled multi-speciality team with experience of pregnancy in women with SMA.

### SMA type

This current series provides detailed insight into the management of pregnancy in women with SMA. Whilst age at delivery in our series is similar to previous reports [3, 4, 6] at 28 years, our population differs from previous reports in terms of SMA type. Specifically, to our knowledge, this series includes the first reported case of a successful pregnancy in a woman with SMA type-1. Furthermore, SMA type-2 is more prevalent, whereas in previous series, SMA type 3 was more prevalent [2]. It needs to be acknowledged that previously women with the more severe phenotype of type-1 and type-2 may have been counselled against pregnancy. As nutritional and respiratory support care has advanced, more of these higher risk women have been successfully supported through pregnancy.

### Pre-pregnancy counselling

To date, PPC has not been described in the literature for women with SMA. Here we have reported that all but two of our patients received PPC. Of the two that did not, one had an unplanned pregnancy and one only had pre-pregnancy genetic counselling. PPC is well evidenced to be beneficial for women of child-bearing age with other chronic diseases [7, 8], and leads to better maternal and fetal outcomes. PPC should be offered to all women living with SMA as it allows for shared decision making regarding reproductive choices and highly personalised care and advice supporting each woman to prepare for the challenges of pregnancy.

### Monitoring respiratory function

Respiratory muscle function, depending on disease severity, can be preserved but one must be aware that women living with SMA can have chronic respiratory failure, requiring nocturnal NIV to support inspiratory muscle function, and cough assist device to support expiratory muscle function and secretion management [2, 9, 10]. In addition, the restrictive ventilatory defect imposed by respiratory muscle weakness will worsen as pregnancy progresses. The diaphragm becomes increasingly splinted as a consequence of the increasing intra-abdominal pressure of the gravid uterus. Indeed, half of the pregnancies in our case series required an increase in respiratory support mid second trimester until after the baby was delivered. Interestingly, this is in contrast to the observations in the Bencivenga et al. series [11], where they reported no change in respiratory function throughout pregnancy. This highlights the much higher risk patients in our series.

In pregnant people with such severe neuromuscular disease, monitoring and surveillance of daytime symptom burden and overnight respiratory status is essential. In line with the low incidence of such pregnant patients, there are no validated respiratory or sleep questionnaires. Standard simple physiological monitoring with spirometry has limited clinical value as often these patients have a significantly reduced vital capacity (VC) due to respiratory muscle weakness and the associated restrictive ventilatory defect, for example VC of less than 1L. This, in essence, means that tracking VC lacks both sensitivity or specificity to have clinical value.

During the 1st and 2nd trimester, we undertook monthly reviews, assessing respiratory and sleep symptoms (e.g. positional breathlessness, breathlessness on hoist transfer, sleep quality, early morning headaches and daytime fatigue) with dual channel overnight oximetry studies to evaluate upper airways obstruction as well as hypoventilation accompanied by daytime spot transcutaneous carbon dioxide and oxygen assessment. During the third trimester, more frequent multi-professional clinical reviews, assessing respiratory and sleep symptoms, took place. These included daytime spot transcutaneous carbon dioxide, as an elevated carbon dioxide level is a warning signal that would necessitate an increase in either the pressure support and/or back up rate in the ventilator. We aimed to maintain carbon dioxide levels within the normal range, or even low normal range, throughout pregnancy. This clinical approach is required to maintain lung volumes, ventilation and gas exchange as pregnancy progresses and intra-abdominal pressure rises and offset the impaired respiratory muscle and diaphragm function.

### Mode of maternal delivery

All women in the current series underwent elective lower segment caesarean section (ELCS) and this should be planned for. This is similar to the findings by Bencivenga et al. [11]. Women with SMA often have significant contractures and positioning them on an operating theatre table is more difficult. We use a combination of gel pads and bean bags to safely support the limbs and the usual lateral supports to ensure a safe left lateral position. In some cases, access to the lower abdomen for a transverse suprapubic incision is complicated and the usual surgical stance to perform the surgery needs to be reconsidered. The surgery itself needs to be gentle with sharp dissection abdominal entry, without the typical stretching of the abdominal wall incision routinely practiced by many obstetricians, to avoid extension of the incision due to tissue fragility. Future contraceptive issues, including tubal ligation, will have been discussed preoperatively, as well as menstrual issues that could be addressed by insertion of a progestogen-releasing intrauterine system at the time of surgery.

Closure of the rectus sheath with a suitable suture is important. The rectus sheath is usually thin and fragile and use of a non-absorbable nylon suture or long term absorbable PDS (Polydioxanone) suture is required. There is usually very little fat, but a 2-layer fat closure with 2/0 monocryl (poliglecaprone 25) is preferred. Skin closure is with subcuticular monocryl and with dermabond (2-Octyl cyanoacrylate) skin adhesive for additional support, which also acts as a dressing. Standard antibiotic prophylaxis is used to prevent infection as are routine uterotonics to prevent uterine atony and post-partum bleeding.

### Mode of anaesthesia

Regional anaesthesia, in the form of intrathecal local anaesthetic and opioid injected in the lumbar region below the termination of the spinal cord, is the preferred mode of anaesthesia for caesarean section in the obstetric population.

This technique may be difficult in the presence of scoliosis, if bony landmarks are distorted, and in the presence of lumbar metal rod fixation, where needle access to the lumbar neuraxium may not be possible.

**Fig. 1:**
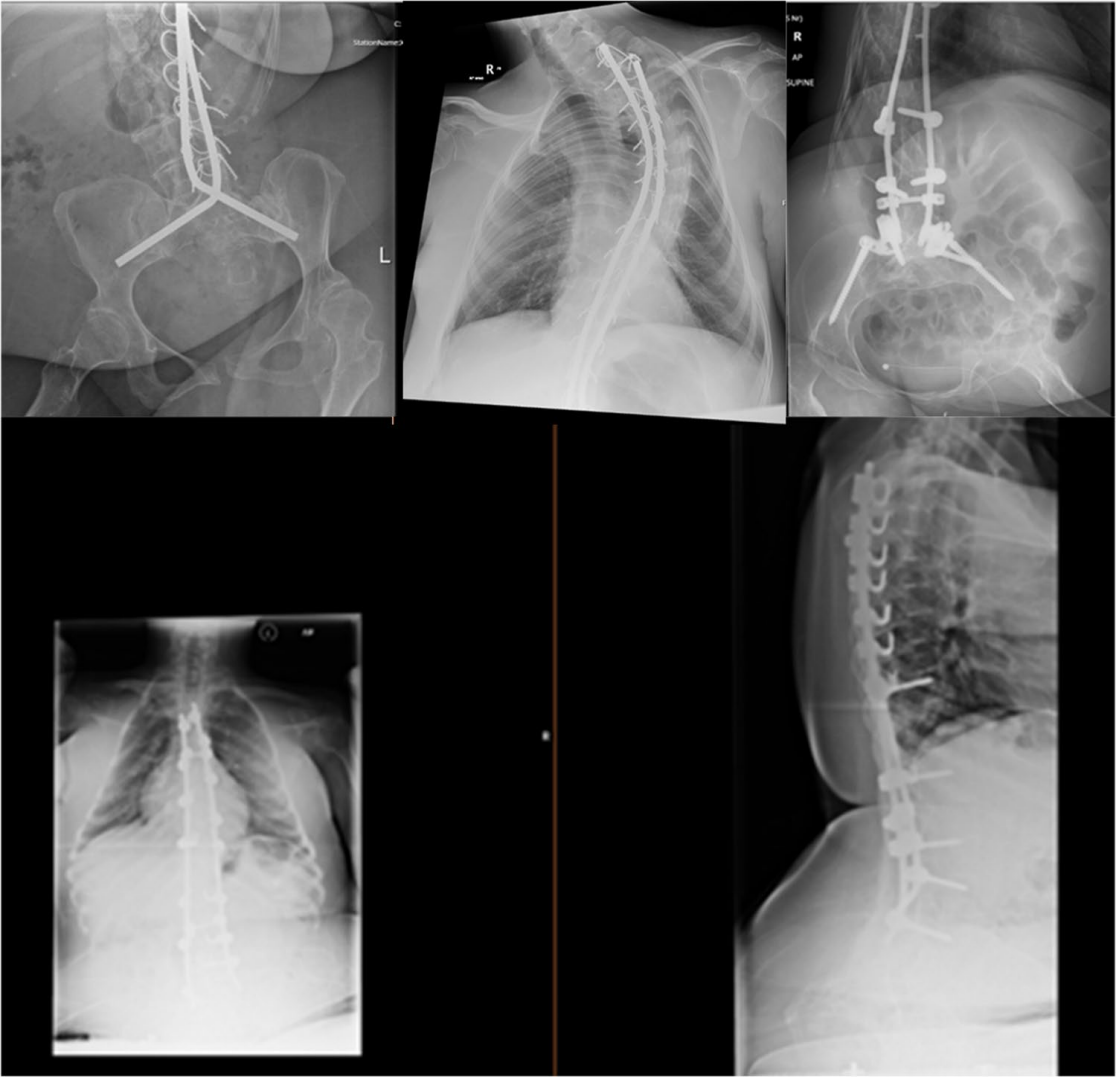
Lumbar spine x-ray images of five of the patients who had spinal fixation prior to pregnancy

**Fig. 2:**
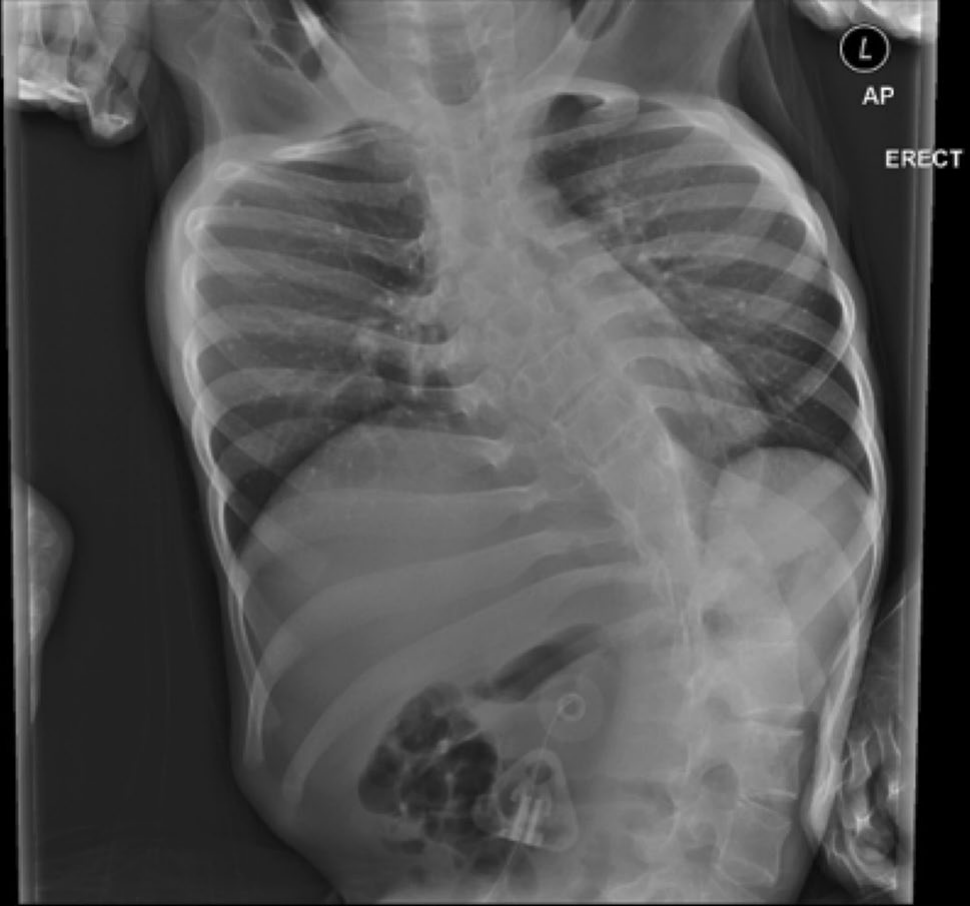
Lumbar spine x-ray image of patient with SMA Type-1 who has severe kyphoscoliosis

SMA types 1 and 2 lead to scoliosis due to the inability of weak spinal muscles to support growing spinal bones. In our case series all patients had severe scoliosis and five had required extensive spinal metal rod fixation involving the lumbar spine (Fig. 1). Our patient with SMA Type-1 had not had spinal fixation but had severe kyphoscoliosis (Fig. 2).

In our case series of severely affected individuals, 100% required general anaesthesia, which contrasts with Bencivenga’s case series [11] in which 80% received regional anaesthesia and 20% general anaesthesia.

One patient consented to an attempt at administering spinal anaesthesia, but unfortunately, this was unsuccessful and a general anaesthetic was given; in her second pregnancy this patient chose not to re-attempt regional anaesthesia.

All patients in our series had required general anaesthesia for other treatments prior to delivery, and as a result, three patients in our series were known to be difficult to intubate; these three patients received awake fibre-optic intubation using infusions of Remifentanil and Propofol to maintain anaesthesia. Where neuromuscular blockade was required, Rocuronium was used, with the reversal agent Sugammadex administered at the end of surgery to ensure full recovery of respiratory muscle function.

All patients received multimodal analgesia including an initial period of intravenous opioid analgesia in a nurse- or patient-controlled delivery device depending on their strength and ability to press the demand button.

### Post-delivery care

As demonstrated in the current series, the multi-speciality team must be wholly knowledgeable of the potential for (a) extended anaesthetic recovery with muscle weakness e.g. neuromuscular blocking agents, (b) respiratory complications e.g. post-operative respiratory failure, impaired secretion clearance and respiratory infection, and (c) impact of post-operative analgesia e.g. respiratory depression from opiates [12]. The monitoring and management of these women in the intensive care unit or high dependency unit, during the post-operative stage, with judicious use of NIV with respiratory therapist-led use of cough assist devices for secretion clearance, reduces the risk of severe post-operative respiratory complications.

Enhanced post-partum care is essential. Skeletal muscle weakness will impact on the speed of recovery as well as caring for and bonding with the new infant. Therefore, early, regular postnatal multidisciplinary reviews should ensure adequate support is available, and aim to minimise the consequences of a potentially prolonged in-patient stay while the mother recovers from the delivery.

### Maternal outcomes

In this case series, all women and their babies survived to time of writing, but seven of the eight pregnancies experienced complications. Women living with SMA can have a spectrum of impairment, the most common being issues with mobility and respiratory function. Indeed, all six women were wheelchair-dependent pre-pregnancy, suggesting that our cohort encompasses women with more severe type 2 SMA disease. In previous series, a significant number were independently mobile pre-pregnancy as these were predominantly SMA type 3 patients. Two in three of the women in the Bencivenga et al. series were ambulant pre-pregnancy [11]. However, Abati et al.’s systematic review reported that at least one in three women experienced persistent worsening of motor symptoms antenatally [4], and not all recovered mobility to pre-pregnancy levels postnatally.

### Neonatal outcomes

In our series, 4 (50%) babies were admitted to the NICU within one week of birth, all for reasons related to prematurity, and 3 had documented RDS. In all cases, their premature delivery was a consequence of third-trimester deterioration in maternal health due to a more severe pre-pregnancy disease state. By contrast, in Abati et al.’s systematic review, the majority of babies were born healthy. Gestation at delivery was not well documented in all studies, but the largest case series reported an average gestational age of 36 weeks and one day [13], which broadly corresponds with the gestational age at birth in our series (35 weeks and five days). Overall, between 3–15% of babies were born with complications in Abati et al.’s review, the majority being related to prematurity and requiring brief admission to NICU. This again is possibly due to the fact that, in the past, women with more severe disease were less likely to get pregnant. Newborn screening for SMA was not offered to any patients in our series.

### Strengths and limitations

Our case series adds significantly to the literature describing outcomes of pregnancies in women with SMA. It provides an up-to-date report and reflects the improved care for women living with more severe phenotypes of SMA who embark on a pregnancy. Although there are expected potential complications in these women and their babies, these can be managed effectively. Furthermore, this current series provides detailed clinical information on the complications and outcomes which can be discussed during pre-pregnancy counselling.

Limitations in our series include a paucity of precise detail in the degree of deterioration in spirometry in the women who decompensated, although this has limited value when patients are already established on nocturnal NIV. Several of our women had shared care between their local hospital and our tertiary specialist maternity centre, therefore not all complications during pregnancy may have been captured accurately in our records.

None of the women in our case series fell pregnant while using disease-modifying treatments (DMTs) for SMA (such as Nusinersen, Risdiplam, or Onasemnogene Abeparvovec). This is because most of the pregnancies in our series concluded before their widespread availability. We are therefore unable to comment on the management of DMTs during pregnancy, other than to point out that their use during pregnancy—and impact on fetus—is currently the subject of on-going research.

## Conclusion

Guidance on managing pregnancy in women with SMA is limited. As with previous case series, our findings are supportive of pregnancy in women with SMA, with regular reviews and monitoring by an experienced multi-speciality team. In the era of disease modifying treatments, we would further recommend a Neurologist be a member of the MDT to help guide their management around pregnancy. Our series also demonstrates that women with a more severe phenotype of SMA can be successfully supported through pregnancy. Indeed, women living with SMA should not be discouraged from becoming pregnant. While pregnancy is likely to be more complicated than for healthy women, with appropriate counselling and clinical support, the overwhelming evidence is that both mother and baby will have good outcomes.

## References

[1] Lally C, Jones C, Farwell W, Reyna SP, Cook SF, Flanders WD (2017) Indirect estimation of the prevalence of spinal muscular atrophy type I, II, and III in the United States. Orphanet J Rare Dis. 10.1186/s13023-017-0724-z 29183396 10.1186/s13023-017-0724-z PMC5704427

[2] Singh N, Hoffman S, Reddi PP, Singh RN (2021) Spinal muscular atrophy: broad disease spectrum and sex-specific phenotypes. Biochim Biophys Acta Mol Basis Dis. 10.1016/j.bbadis.2020.166063 33412266 10.1016/j.bbadis.2020.166063 PMC7867633

[3] Elsheikh BH, Zhang X, Swoboda KJ, Chelnick S, Reyna SP, Kolb SJ et al (2017) Pregnancy and delivery in women with spinal muscular atrophy. Int J Neurosci. 10.1080/00207454.2017.1281273 28102719 10.1080/00207454.2017.1281273

[4] Abati E, Corti S (2018) Pregnancy outcomes in women with spinal muscular atrophy: a review. J Neurol Sci. 10.1016/j.jns.2018.03.001 29627031 10.1016/j.jns.2018.03.001

[5] Fiona Norwood SRS (2010) 179th ENMC international workshop: pregnancy in women with neuromuscular disorders. Neuromuscular disorders. Naarden, The Netherlands, pp 183–190 10.1016/j.nmd.2011.05.009 21689937

[6] Carter G, Bonekat H, Milio L (1994) Successful pregnancies in the presence of spinal muscular atrophy: two case reports. Arch Phys Med Rehabil 75(2):229–231 8311683

[7] Nana M, Stannard M, Nelson-Piercy C, Williamson C (2023) The impact of preconception counselling on maternal and fetal outcomes in women with chronic medical conditions: a systematic review. Eur J Intern Med. 10.1016/j.ejim.2022.11.003 36435697 10.1016/j.ejim.2022.11.003

[8] Wahabi H, Fayed A, Esmaeil S, Elmorshedy H, Titi M, Amer Y et al (2020) Systematic review and meta-analysis of the effectiveness of pre-pregnancy care for women with diabetes for improving maternal and perinatal outcomes. PLoS ONE. 10.1371/journal.pone.0237571 32810195 10.1371/journal.pone.0237571 PMC7433888

[9] Vicino A, Bello L, Bonanno S, Govoni A, Cerri F, Ferraro M et al (2023) Respiratory function in a large cohort of treatment-naïve adult spinal muscular atrophy patients: a cross-sectional study. Neuromuscul Disord. 10.1016/j.nmd.2023.10.002 37945485 10.1016/j.nmd.2023.10.002

[10] Fiona Norwood SRS (2012) 179th ENMC international workshop: Pregnancy in women with neuromuscular disorders 5–7 November 2010, Naarden. Netherlands Neuromuscular Disorders 22:183–190 21689937 10.1016/j.nmd.2011.05.009

[11] Bencivenga RP, Zoppi D, Russo A, Cassano E, Tozza S, Iodice R et al (2023) Pregnancy experience in women with spinal muscular atrophy: a case series. Acta Myol. 10.36185/2532-1900-316 38090543 10.36185/2532-1900-316 PMC10712658

[12] Awuku K, Younker A, Osei-Bonsu B, Nalbone J, Master A, Li D et al (2024) Anesthetic management of a patient with spinal muscular atrophy type III undergoing emergent caesarean section: a case report. Open J Anesthes. 10.4236/ojanes.2024.146014

[13] Awater C, Zerres K, Rudnik-Schoneborn S (2012) Pregnancy course and outcome in women with hereditary neuromuscular disorders: comparison of obstetric risks in 178 patients. Eur J Obstet Gynecol Reprod Biol. 10.1016/j.ejogrb.2012.02.020 22459654 10.1016/j.ejogrb.2012.02.020

